# Growth arrest and forced differentiation of human primary glioblastoma multiforme by a novel small molecule

**DOI:** 10.1038/srep05546

**Published:** 2014-07-03

**Authors:** Tae-Wook Kang, Soon Won Choi, Se-Ran Yang, Tae-Hoon Shin, Hyung-Sik Kim, Kyung-Rok Yu, In-Sun Hong, Seonggu Ro, Joong Myung Cho, Kyung-Sun Kang

**Affiliations:** 1 Adult Stem Cell Research Center, College of Veterinary Medicine, Seoul National University, Seoul, Republic of Korea; 2 BK21 PLUS Program for Creative Veterinary Science Research, Research Institute for Veterinary Science and College of Veterinary Medicine, Seoul National University; 3 Department of Thoracic and Cardiovascular Surgery, Kangwon National University Hospital, School of Medicine, Kangwon National University, Chuncheon, Republic of Korea; 4 Department of Molecular Medicine, Gachon University, Incheon, Republic of Korea; 5 Lee Gil Ya Cancer and Diabetes Institute, Gachon University, Incheon, Republic of Korea; 6 CrystalGenomics, Inc., Korea Bio Park, 700 Daewangpangyo-ro, Bundang-gu, Seongnam, Republic of Korea; 7 These authors contributed equally to this work.

## Abstract

Glioblastoma multiforme is the most common malignant brain tumor in adults, with an average survival of less than one year due to its resistance to therapy. Recent studies reported that GBM initiates from CD133-expressing cancer stem cells (CSC). However, the efficacy of CSC targeting is limited. A newly developed approach in cancer treatment is the forced differentiation of cancer cells. Here, we show that the treatment of the novel small molecule, CG500354, into CD133-expressing human primary GBM cells induces growth arrest by cell cycle regulators, p53, p21, p27 and phase-specific cyclins, and neural differentiation, as confirmed by neural progenitor/precursor markers, nestin, GFAP and Tuj1. When GBM-derived cells caused the tumors in NOD/SCID mice, CG500354 induced GBM-derived cells differentiation into Tuj1 and GFAP expressing cells. We next demonstrated that CG500354 plays a tumor-suppressive role via cAMP/CREB signaling pathway. CG500354 increases not only the extracellular cAMP level but also the protein level of PKA and CREB. Additionally, both mimetic substances, Forskolin and Rolipram, revealed comparable results with CG500354. Our findings indicate that induction of growth arrest and neural differentiation via cAMP/CREB signaling pathway by CG500354 treatment suggests the novel targeting of PDE4D in the development of new drugs for brain tumor therapy.

GBM is the most common lethal primary brain tumor in adults, with a median survival of less than 12 months due to its radioresistance and chemoresistance^1^,^2^,^3^. It has recently been accepted that undifferentiated tumor cells, called CSCs, in various tissues play a pivotal role in the initiation and progression of cancers^4^. CSCs comprise only a small portion of the tumor, and each single cell can give rise to a new tumor. Regarding the biological properties of CSCs, recent evidence has emerged that CSCs are similar to tissue-specific stem cells with respect to self-renewal and multi-lineage differentiation capacity, but they differ in their long-term proliferative potential. This uncontrolled renewal potential of CSCs might be the reason for tumor relapse after conventional cancer therapy.

Like tissue-specific stem cells, there are no universal biomarkers for CSCs. Nonetheless, the cell surface marker CD133 has been frequently applied for the identification of tissue-specific stem cells. Over many years, the expression of CD133 has been detected in various stem/progenitor cells, particularly in cells of the human neural systems, including the fetal brain, the post-mortem retina and embryonic stem cell-derived neural progenitors^5^,^6^,^7^. Additionally, CD133 has been most frequently used as a putative biomarker of CSCs in brain tumors^8^. Recent studies have suggested that a GBM subpopulation expresses CD133 and is enriched for CSCs^1^,^9^,^10^,^11^. This subpopulation shows an increased tumorigenic potential than subpopulations that are devoid of CD133 expression^12^,^13^,^14^,^15^. Moreover, a reliable study demonstrated that the CSC population could be targeted in GBM therapy^16^. There have been many attempts to develop targeted therapies of tumorigenic cell populations, but an effective therapy has not yet been achieved.

Apart from eradication the CSC population, the limitation of tumor growth, which can be realized by forcing the tumor cells to differentiate, is a new concept in the search for alternative cancer therapies. Piccirillo and colleagues have demonstrated that bone morphogenetic protein 4 (BMP4) induces the neural differentiation of human GBM-derived cells. They showed that BMP4 exerts growth inhibitory effects on CD133-expressing GBM-derived cells *in vitro* and that BMP4 treatment hinders tumorigenicity *in vivo*^17^. Other studies have demonstrated that a combination of retinoic acid and arsenic trioxide strongly promotes the terminal differentiation of leukemic cells and that cAMP is involved in the depletion of the leukemic CSCs using both drugs^18^,^19^,^20^. Although multiple drugs were found to affect the phenotype of the tumor mass, the efficacy of these drugs was limited and did not affect the tumor-initiating cells.

A few studies have investigated whether phosphodiesterase 4 (PDE4) plays a crucial role in tumor suppression^21^,^22^,^23^. As it is well known that PDE4 reduces the level of cAMP within cells, PDE4 inhibitors have been frequently applied to promote cAMP/CREB signaling pathway. The cAMP/CREB signaling pathway is involved in many biological functions and appears to play a role in differentiation^24^,^25^. Furthermore, cAMP/CREB signaling pathway can induce the differentiation of human cancer cells and mesenchymal stem cells^26^,^27^,^28^. In tumor biology, it has been demonstrated that genes related to the cAMP/CREB signaling pathway act as oncogenes or oncogene suppressors^29^,^30^,^31^,^32^,^33^. Rolipram, a specific PDE4 inhibitor, is commonly known as a memory enhancer^34^,^35^ due to its role in increasing the level of cAMP and the phosphorylation of CREB^36^,^37^. Rolipram was recently used in a GBM study, owing to its promoting cAMP signal transduction^38^. The Forskolin-induced increase of cAMP could leads to up-regulation of gap junctional intercellular communication, which is usually inhibited in cancer cells, and to neuronal differentiation of SVG cells^39^. Although recent reports have indicated the significant role of cAMP/CREB signaling pathway in the differentiation of cancer cells, the cellular mechanism by which cAMP and CREB force the differentiation process remains to be unveiled.

The relationship between tumor suppression and neural differentiation in GBM has been reported in several studies, however the tumor-suppressive effects of PDE4D inhibitors have rarely been studied. There are four PDE4 subfamilies (A to D), which are comprised of more than 20 different isoforms^38^ that regulate the cellular cAMP level through their hydrolytic activity^40^,^41^. It is now clearly known yet the variants of PDE4 and used for an anti-cancer effect out of more than 20 PDE variants. However, CG500354, which targets the specific variant PDE4D, may provide a closer step in solving the therapeutic issues of aggressive glioblastoma.

In this work, we suggest that the novel small molecule, CG500354, plays a tumor-suppressive role in human GBM by the increased expression of p53, which subsequently induces GBM growth arrest. We determined that CG500354 forces the neural differentiation of GBM-derived cells by regulating cAMP/CREB signaling pathway. In particular, we demonstrated that the tumor suppressors, Rolipram and Forskolin, are both involved in cAMP/CREB signaling pathway and can induce the differentiation of GBM-derived cells. Thus, our data reveal the anti-cancer effects of the novel small molecule CG500354 and its specific mechanism by which this molecule increases p53 expression and cAMP/CREB activity.

## Results

### CG500354 rapidly induces differentiation in human primary GBM-derived neurospheres without producing cytotoxic effects

**Figure 1:**
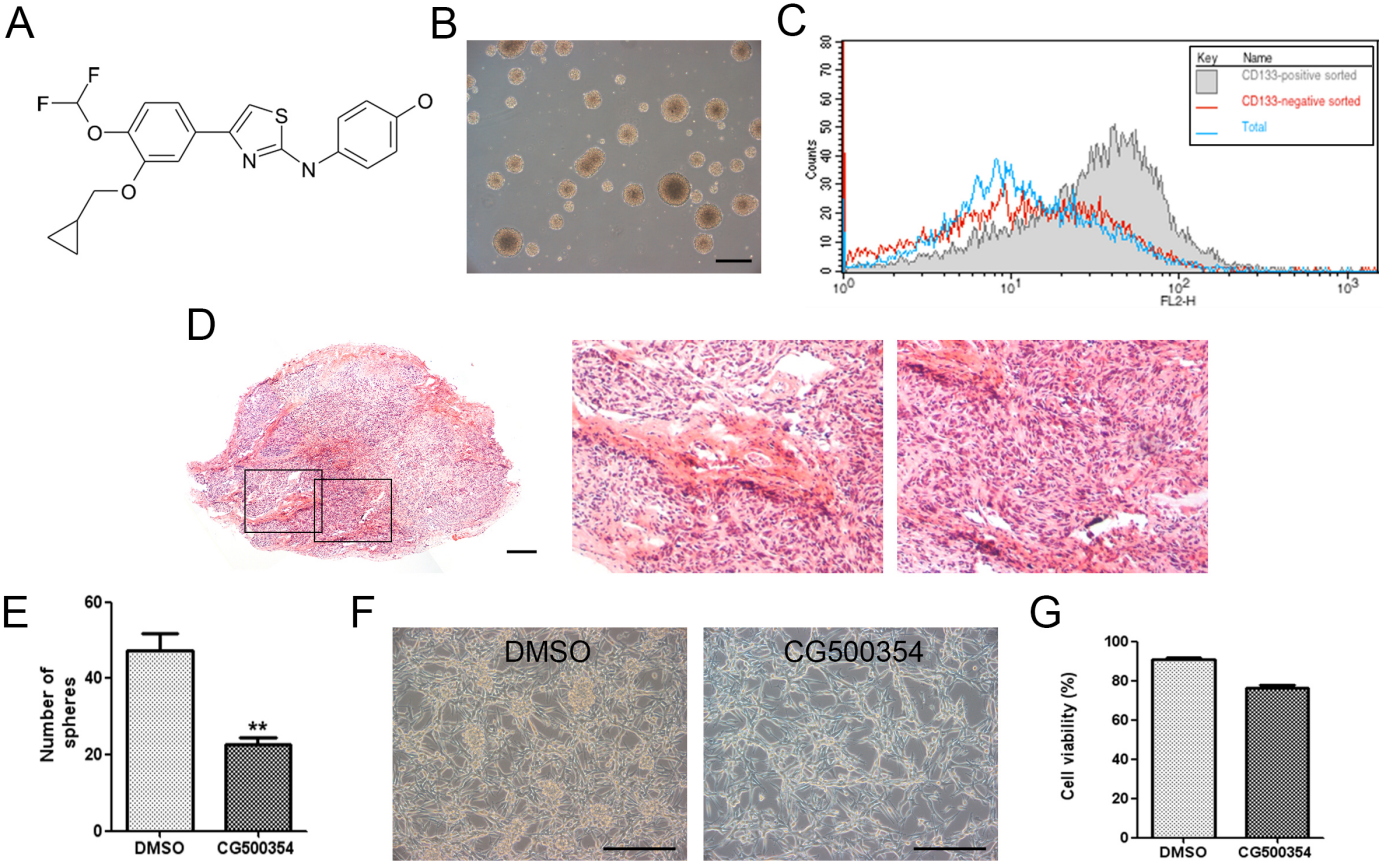
Characterization of human primary GBM-derived cells. (A) Chemical structure of the small molecule CG500354. (B) Representative phase-contrast image of GBM-derived spheres in non-adherent culture conditions. Scale bar = 1 mm. (C) The GBM-derived cells were characterized by flow cytometry after sorting the CD133-positive cell population using magnetic-activated cell sorting. (D) A glioblastoma (left) from the GBM-derived cells in a xenograft mouse model has been characterized with pseudopalisading necrosis (middle) and endothelial proliferation (right) after hematoxylin and eosin staining. Scale bar = 400 μm. (E) CG500354 significantly reduced the number of spheres after 72 hours of exposure in a clonogenic assay. (F) Representative phase-contrast images of GBM-derived cells treated with DMSO or CG500354. Scale bar = 1 mm. (G) A trypan blue exclusion test was performed to calculate the number of viable cells compared to the total cell number in the GBM-derived cell population after 72 hours of treatment with CG500354 (3 μM) or DMSO. DMSO was used as a vehicle control.

We first determined the effects of a novel small molecule, CG500354, on the oncogenic phenotype of human primary GBM-derived cells. CG500354 has been designed, synthesized and evaluated by CrystalGenomics, Inc. using its structure-based drug discovery platform which employs the integrated technologies of the SPS™ (Soluble Protein Solution), technology for obtaining soluble forms of disease related proteins that are usually insoluble when over-expressed in heterologous systems, SCP™ (Structural Chemo Proteomics), technology for rapidly generating novel leads using the 3-D structure information of the target proteins, and SDF™ (Structural-based Drug Factory), technology to optimize novel leads to drug candidate compounds using the 3-D structure information of the complexes of target proteins and inhibitors. The synthetic compound, CG500354, is an inhibitor of cAMP-specific 3′,5′-cyclic phosphodiesterase 4D (PDE4D), which plays a role in cAMP homeostasis by hydrolyzing the second messenger cAMP (Fig. 1A). Human primary cells from three different GBM origins, GBM559, GBM592 and GBM626, were grown as neurospheres under non-adherent culture conditions (Fig. 1B). A very small portion of human GBM expresses CD133, which is a typical marker of neural stem cells. It is known that this fraction plays an important role in the proliferation and survival of GBM. To classify our GBM sample, we separated the CD133 population from the GBM-derived cells using magnetic-activated cell sorting (MACS). Then, we performed flow cytometry to detect the CD133-positive GBM-derived cells and identified the present of CD133-expressing subpopulation (Fig. 1C). The GBM-derived cells were able to form a glioblastoma in a xenograft mouse model. This brain tumor showed two typical histopathological features of GBM: pseudopalisading necrosis and endothelial proliferation (Fig. 1D).

To identify the effects of CG500354, we cultured GBM-derived neurospheres in non-adherent culture conditions and performed a clonogenic assay after 72 hours of treatment with CG500354 or dimethyl sulfoxide (DMSO) as a vehicle control. In clonogenic assay, the CG500354-treated neurospheres exhibited the decreased size in contrast to the DMSO-treated neurospheres (Fig. 1E). While we seeded neurospheres on the PLO/FN-coated dishes for immunocytochemistry, we could observed that most CG500354-treated neurospheres turn into adherent and differentiated state, but many DMSO-treated neurospheres still remain as a sphere (Fig. 1F). However, in viability assay, number of GBM cells was not prominently changed in response to CG500354 (Fig. 1G). These results suggest that the small molecule, CG500354, induces a dramatic change in the morphology of human GBM-derived neurospheres without cellular cytotoxicity.

### CG500354 attenuates the clonogenic potential of GBM-derived cell populations via up-regulation of p53

**Figure 2:**
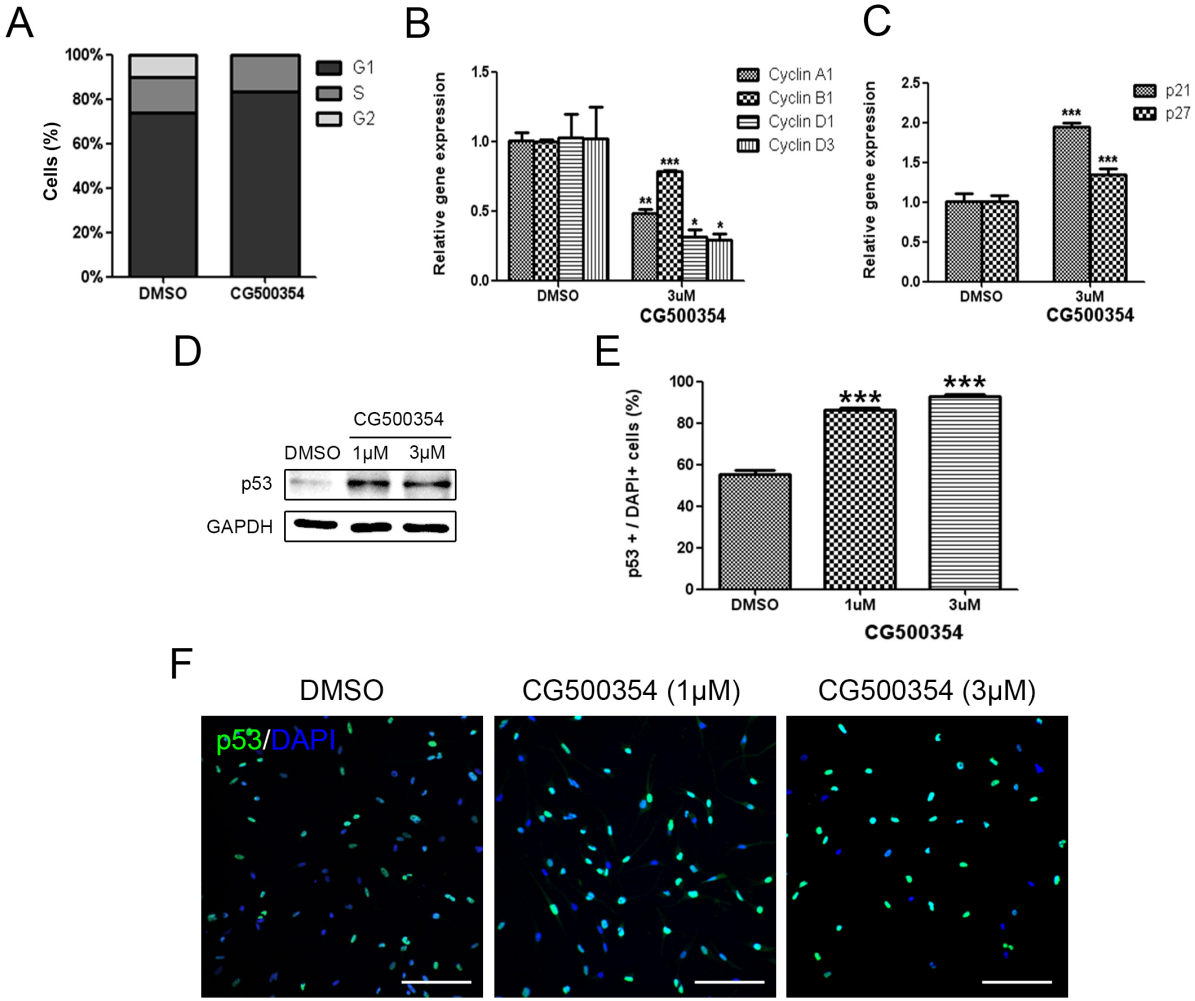
Growth arrest of human GBM via CG500354-mediated p53 overexpression. (A) Cell cycle analysis shows that the percent of cells in G1 phase was increased after CG500354 treatment. (B–C) Quantitative RT-PCR analysis shows that the cell cycle regulators cyclin A1, B1, D1 and D3 were significantly down-regulated and that the Cdks p21 and p27 were significantly up-regulated in the presence of CG500354. (D) Western blot analysis shows that p53 expression was increased by treating the cells with 1 μM or 3 μM CG500354. The full-length blots are included in the supplementary information (Suppl. Fig. S5). (E–F) GBM-derived cells treated with CG500354 were stained with a p53 antibody. The number of p53-expressing cells (green) were significantly increased after CG500354 treatment at both concentrations (1 μM and 3 μM). Nuclei (blue) were counterstained with DAPI. Scale bar = 0.5 mm.

To determine the inhibitory effects of CG500354 on the growth of human primary GBM-derived neurospheres, we performed a cell cycle analysis. In the presence of CG500354, the G1 fraction of the total neurosphere cells was increased from 73.6% to 83.3%, whereas cells were not found in the G2 fraction (Fig. 2A). As additional evidence of the cell cycle arrest, we performed a quantitative RT-PCR analysis. Cell cycle regulators compose a complex regulatory system and include cyclins and cyclin-dependent kinases (Cdks). Cdks are inactivated unless bound to phase-specific cyclins. Here, we assessed the expression of cyclin A1 for S phase, cyclin B1 for M phase and cyclin D1 and D3 for G1 phase. The mRNA levels of these four cyclins were significantly decreased in the presence of CG500354 (Fig. 2B). We further analyzed the gene expression level of two Cdk inhibitor proteins, p21 and p27, and found that both were significantly up-regulated (1.95-fold and 1.35-fold, respectively) in CG500354-treated GBM-derived neurospheres (Fig. 2C). These results indicate that CG500354 induces growth arrest in GBM-derived neurospheres by inhibiting cell cycle regulators.

The tumor suppressor protein p53 is a transcription factor that has crucial effects on cell cycle arrest, DNA repair, apoptosis, senescence and angiogenesis^42^,^43^. Thus, we analyzed p53 and its pro-apoptotic targets following CG500354 treatment. In our results, CG500354 treatment with 1 μM and 3 μM increased the protein expression level of p53 in the GBM-derived neurospheres (Fig. 2D). In immunocytochemistry, CG500354 treatment increased the number of p53-expressing cells from 55.2% to 86.3% by 1 μM CG500354 and to 93.1% by 3 μM CG500354 (Fig. 2E–F). Western blot analysis shows increased expression level of pro-apoptotic targets, BAK and BAX (Suppl. Fig. S1). These data demonstrated that the growth arrest was triggered by p53 and its downstream targets, such as p21, p27 and pro-apoptotic targets, whereas the forced neural differentiation was induced by cAMP/CREB signaling pathway.

### CG500354 induces the neural differentiation of GBM-derived cells

**Figure 3:**
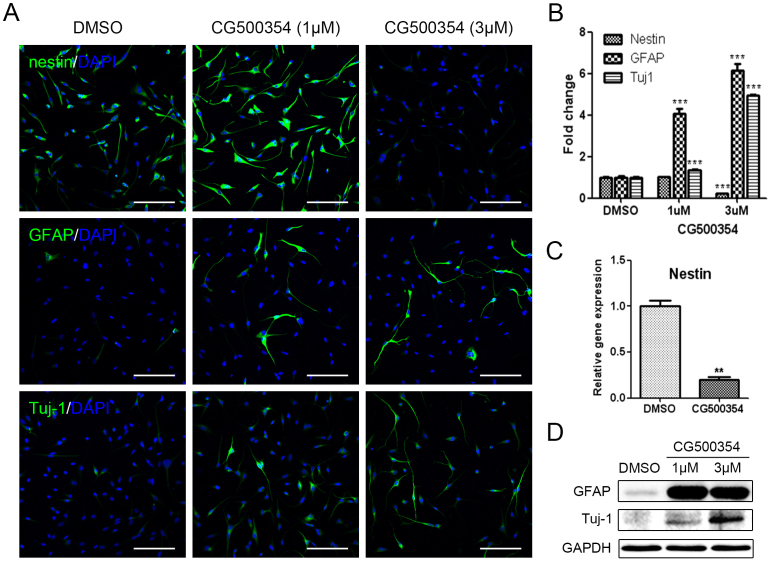
Neural differentiation of human GBM upon CG500354 treatment. (A–B) Representative immunochemical images of primary GBM-derived cells show that CG500354 treatment decreased the number of nestin-positive neural progenitor cells and increased the number of GFAP- and Tuj1-positive neural subtypes. The effect of CG500354 on the neural differentiation of GBM was more effective with 3 μM than 1 μM CG500354. Nuclei (blue) were counterstained with DAPI. Scale bar = 0.5 mm. (C) Quantitative RT-PCR analysis shows that the relative expression level of nestin was significantly reduced by 3 μM CG500354. (D) Western blot analysis shows that the expression of the GFAP and Tuj1 proteins was up-regulated by CG500354 treatment. The full-length blots are included in the supplementary information (Suppl. Fig. S6).

To identify the effects of CG500354 on the neural differentiation of human primary GBM-derived cells, 2 × 10^4^ cells were seeded in 24-well plates containing PLO/FN-coated coverslips and treated with CG500354 at a concentration of 1 μM or 3 μM. We subsequently examined the expression of the neural progenitor cell marker nestin, the neuronal marker Tuj1 and the astrocyte marker GFAP in the GBM-derived cells after CG500354 treatment. After 72 hours, the cells exhibited the neuronal morphology and were immunocytochemically stained (Fig. 3A). In the vehicle control, 92.4% of the cells expressed nestin, and this percentage was significantly reduced to 20.3% after 3 μM CG500354 treatment, whereas 1 μM CG500354 did not affect nestin expression (Fig. 3B). Analysis of nestin expression at the mRNA level showed identical results (Fig. 3C). In opposition to nestin, the number of GFAP- and Tuj1-expressing cells was significantly increased upon CG500354 treatment. In comparison to the vehicle control, the cell numbers rose up to 6-fold in GFAP and 5-fold in Tuj1 with 3 μM CG500354 (21.4% and 79.6%, respectively). In addition, western blot analysis showed similar expressing pattern of GFAP and Tuj1 with immunocytochemistry (Fig. 3D). We observed that the neural progenitor/precursor marker expression was changed in a dose-dependent manner. From the above, the concentration of 3 μM CG500354 was most efficient in the neural differentiation of human GBM-derived cells. Altogether, these results indicate that CG500354 induces the neural differentiation with GFAP and Tuj1 up-regulations.

### Activation of the cAMP/CREB signaling pathway by using CG500354 leads to growth arrest in GBM-derived cells

**Figure 4:**
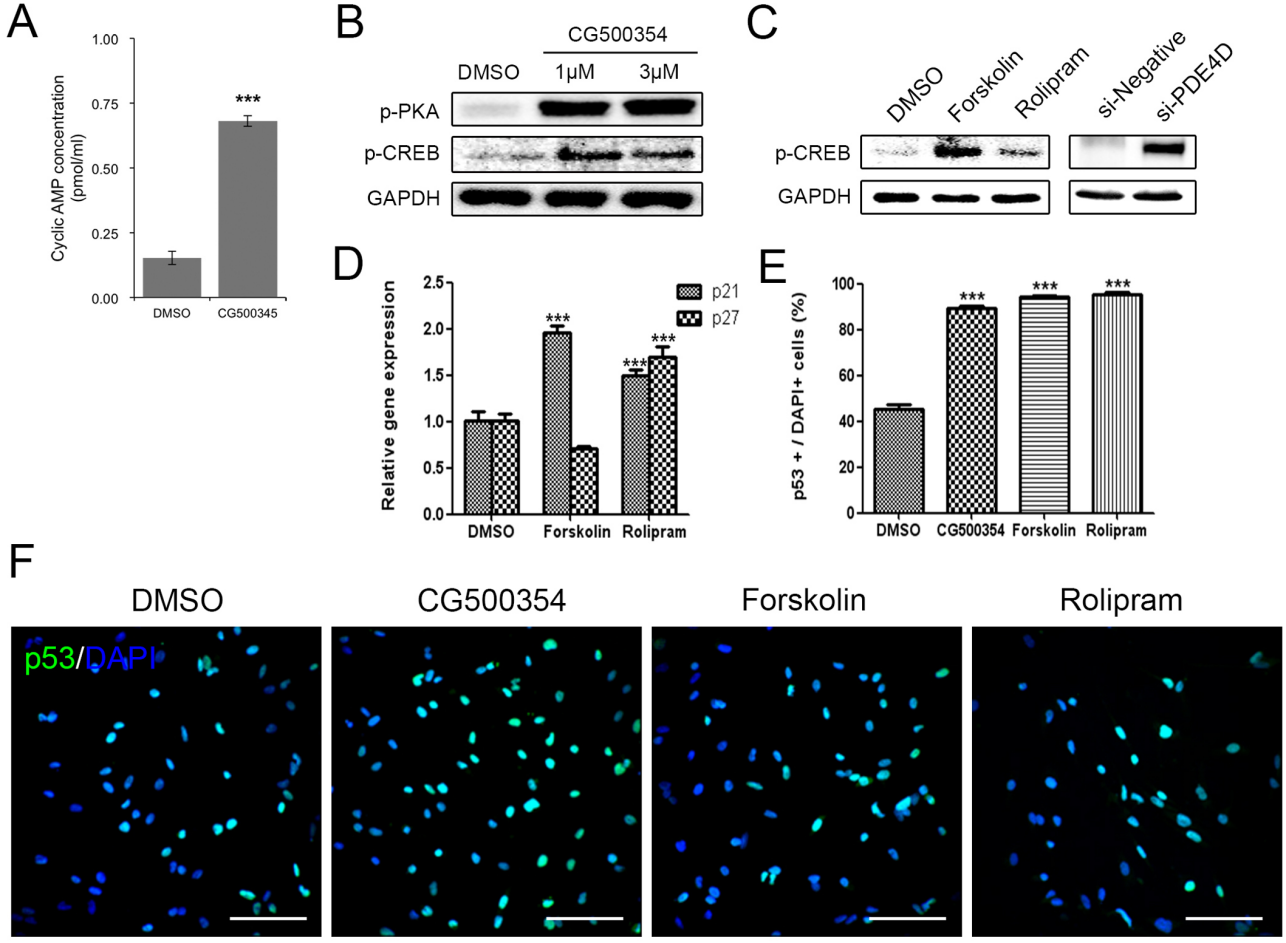
Stimulation of cAMP/CREB signaling pathway by CG500354 and mimetic substances. (A) Measurement of the extracellular cAMP level was performed with a Cyclic AMP EIA Kit. The supernatant from the CG500354-treated GBM cells showed a significantly higher level of cAMP than that from the DMSO-treated GBM cells. (B) Western blot analysis shows that the expression of phosphorylated PKA and CREB was strongly induced by CG500354 treatment. The full-length blots are included in the supplementary information (Suppl. Fig. S7). (C) Both mimetic substances (Forskolin and Rolipram) and si-PDE4D also increased the expression of phosphorylated CREB. The full-length blots are included in the supplementary information (Suppl. Fig. S8). (D) Quantitative RT-PCR analysis reveals that both mimetic substances induced p21 expression and only Rolipram also increased p27 expression. (E–F) GBM-derived cells treated with CG500354, Forskolin or Rolipram were stained with a p53 antibody. The numbers of p53-expressing cells (green) in the three groups were all equivalently high. Nuclei (blue) were counterstained with DAPI. Scale bar = 0.5 mm.

As previously described, CG500354 is an inhibitor of PDE4D, which regulates the cAMP signaling. In the cAMP/CREB signaling pathway, the accumulation of cAMP induces the diffusion of a subunit of cAMP-dependent phosphorylated protein kinase A (PKA) into the nucleus and activates cAMP response element-binding protein (CREB). In several studies, CREB is described to stimulate the differentiation of human cancer cells and mesenchymal stem cells^26^,^27^,^28^. Here, we examined the CG500354-mediated activation of the cAMP/CREB signaling pathway. After CG500354 treatment, the supernatants from the cell culture of GBM cells were harvested and measured the secreted cAMP concentration. These measurements revealed an approximately 4.5-fold increase in the level of cAMP secretion in the CG500354-treated GBM-derived cells compared to the control cells (Fig. 4A). We then performed western blot analysis of the major downstream targets PKA and CREB to verify the activation of the cAMP signaling pathway. CG500354 treatment effectively increased the expression of both phosphorylated PKA and CREB, and this up-regulation was detected from both CG500354 doses (Fig. 4B).

To compare the potential of CG500354 in GBM with two known mimetic substances, we treated GBM-derived cells with the cAMP regulator Forskolin and the PDE4 inhibitor Rolipram. Then, we confirmed both mimetic substances increased the level of phosphorylated CREB as CG500354 (Fig. 4C). Quantification of the western blot analysis showed that the levels of phosphorylated CREB in the Forskolin-treated cells were approximately 11-fold higher than that in the control cells, but not as much as CG500354. In addition, we performed the direct suppression of PDE4D expression using a siRNA, targeting the PDE4D gene (si-PDE4D). This si-PDE4D reduced 73.6% of the PDE4D expression and increased the level of phosphorylated CREB in the human primary GBM-derived cells as expected (Suppl. Fig. S2 and Fig. 4C).

To explore whether these two mimetic substances can also induce growth arrest like CG500354, we treated GBM-derived cells with CG500354, Forskolin or Rolipram for 72 hours, and the treated GBM-derived cells were analyzed using immunocytochemistry (Fig. 4E–F). Similar to the CG500354-treated cells, the Forskolin- and Rolipram-treated cells showed significant increases in p53 protein expression. The percentages of p53-expressing cells in both of the groups treated with mimetic substances were as high as that observed in the group treated with CG500354. Moreover, treatment with Forskolin and Rolipram induced the gene expression of the p53 downstream targets p21 and p27 (Fig. 4D). The Rolipram significantly increased the expression of both p21 and p27, which inhibit cell cycle progression. Therefore, CG500354 and its mimetic substances Forskolin and Rolipram are able to induce growth arrest in GBM-derived cells. From the analyses of cAMP signaling and cell cycle regulators, we concluded that CG500354 appeared more effective than both mimetic substances and it takes a novel therapeutic potential to handle the GBM growth arrest.

### Mimetic substances, including si-PDE4D, mimic the effect of CG500354 on the neural differentiation of GBM-derived cells

**Figure 5:**
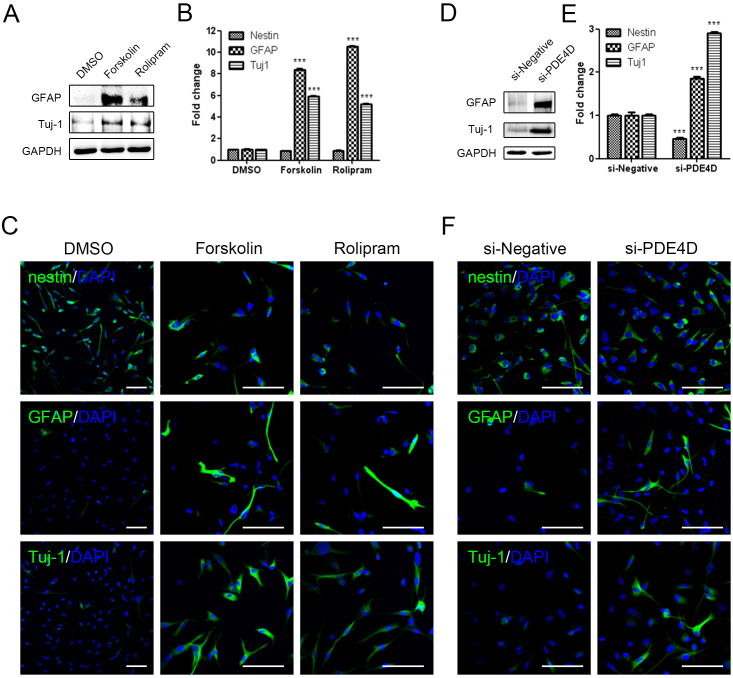
Neural differentiation of human GBM with mimetic substances and si-PDE4D. (A), (D) Western blot analysis shows that the expression of the neural subtype markers GFAP and Tuj1 was upregulated upon Forskolin, Rolipram and si-PDE4D. The full-length blots are included in the supplementary information (Suppl. Fig. S9–S10). (B–C), (E–F) Representative immunochemical images of primary GBM-derived cells reveal that Forskolin, Rolipram and si-PDE4D induced an increase in the number of the GFAP- and Tuj1-positive cells. Upon Forskolin or Rolipram treatment, the nestin-expressing cell population did not decrease (B), whereas this population was reduced by si-PDE4D as expected (E). Nuclei (blue) were counterstained with DAPI. Scale bar = 0.5 mm.

We next investigated the effect of these mimetic substances on the neural differentiation in human primary GBM-derived cells. After Forskolin and Rolipram treatment, the neural differentiation markers GFAP and Tuj1 showed the up-regulation in the western blot analysis (Fig. 5A). We further performed the immunocytochemistry to analyze the nestin, GFAP and Tuj1 expressions (Fig. 5B–C). Similar to CG500354, Forskolin- and Rolipram-treatment cells were detected 8 to 10-fold more GFAP-expressing cells and 5-fold more Tuj1-expressing cells in comparison to the vehicle control. Unlike CG500354, the number of nestin-expressing cells did not change after treatments of two mimetic substances. These results suggest that Forskolin and Rolipram induce the differentiation of GBM-derived cells but do not deplete the cell population remaining in the neural progenitor state.

To further investigate the neural differentiation phenomena induced by CG500354, we performed gene silencing of PED4D using siRNA and it induced the overexpression of GFAP and Tuj1 in the GBM-derived cells as expected. The protein expression levels of GFAP and Tuj1 were significantly up-regulated in the si-PDE4D-transfected GBM-derived cells (Fig. 5D). Moreover, the immunocytochemical analysis showed that the decease of nestin-expression and increase of GFAP- and Tuj1-expression (Fig. 5E–F). Both our immunocytochemistry and western blot analyses revealed that si-PDE4D affects the expression of neural differentiation markers similar to CG500354. From the above, we could indicate that novel CG500354 have shown higher potential in cell cycle regulation than Rolipram while activating cAMP signaling pathway as Forskolin. Therefore, our data on the mimetic substances and si-PDE4D provide direct evidence that CG500354 works as a multi-controller that induces neural differentiation and growth arrest in human primary GBM-derived cells.

### *In vivo* neural differentiation of GBM-derived cells is induced by CG500354 treatment

**Figure 6:**
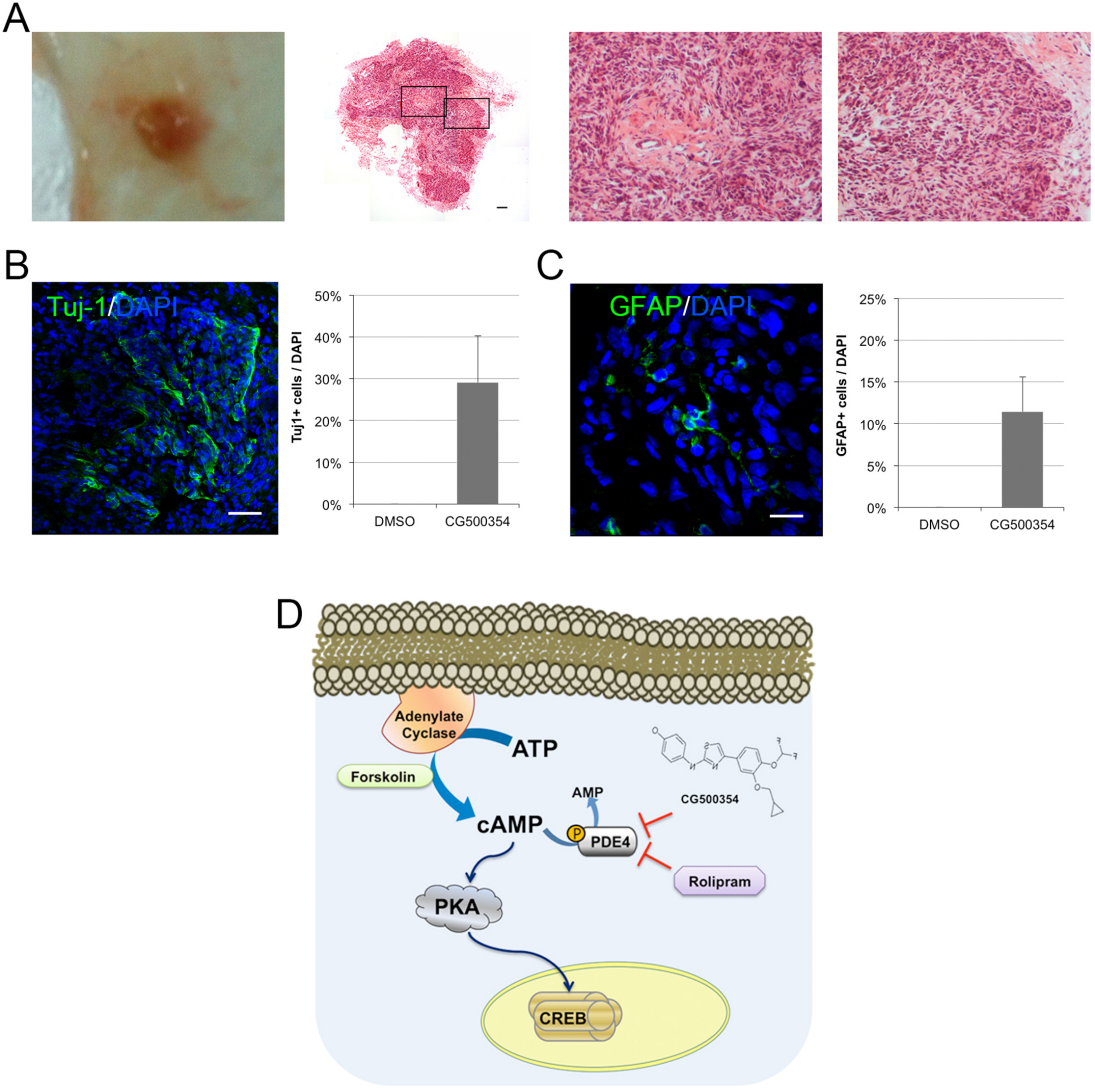
*In vivo* neural differentiation of human primary GBM-derived cells after xenotransplantation. (A) After GBM tumor formation, tumors were isolated and stained with hematoxylin and eosin. A composited image (second from left) reveals an entire section of a tumor and two high-resolution images indicates pseudopalisading necrosis (second from right) and endothelial proliferation (right). (B–C) Representative immunochemical images of brain sections from GBM-derived tumors show that the cells inside of the tumors were forced to differentiate into Tuj1- and GFAP-expressing neural subtypes. (D) Schematic diagram of the mechanism of CG500354-triggered cAMP/CREB signaling pathway. Likewise, both of the mimetic substances Forskolin and Rolipram are involved in this signal transduction pathway.

To validate the *in vivo* effects of CG500354, we performed subcutaneous xenotransplantation of GBM-derived cells into NOD/SCID mice. After GBM tumor formation, we treated mice with CG500354 or DMSO via intraperitoneal injection for 10 days. Then, we sacrificed the mice and isolated GBM tumors from the host for hematoxylin and eosin staining (Fig. 6A). These GBM tumors were characterized with pseudopalisading necrosis, endothelial proliferation and irregular nuclear contours. Most part of the tumor showed a small nuclear size and 29.1% of this part appeared to be Tuj1-positive (Fig. 6B). But, the other part of the tumor showed a large nuclear size and 11.4% of this part appeared to be GFAP-positive by immunohistochemistry (Fig. 6C). These results indicated that approximately 40% of the GBM tumor was induced to differentiate into neural subtypes by treating CG500354, a novel small molecule.

## Discussion

In this study, we investigated the dual effects of CG500354 as a PDE4D inhibitor on human primary GBM. First, we showed that CG500354 induces growth arrest and attenuates stemness in GBM-derived cells. In human GBM cells, this small molecule regulates the expression of the tumor suppressor p53 and its downstream target p21 as well as p27. The increased expression of both Cdk inhibitors led to the decreased expression of a number of phase-specific cyclins and a reduction in the clonogenic potential of the GBM-derived cells. Second, CG500354 accelerated the neural differentiation, which were expressed Tuj-1 and GFAP of GBM-derived cells. Our findings suggest that the cAMP/CREB signaling pathway is involved in this neural differentiation via the phosphorylation of PKA and CREB following the CG500354-mediated up-regulation of cAMP.

In the preliminary study, we have done all experiments with three different GBM origins, including GBM559, GBM592 and GBM626, to analyze CG500354 effects. Those three GBM origin-derived cell types have shown similar results. Coincidently, a quantitative RT-PCR analysis showed reduced gene expressions of SOX2 and nestin and an increased gene expression of p53 in GBM559-, GBM592- and GBM626-derived cells (Suppl. Fig. S3). We have representatively shown the results of GBM559 in this study.

Recent studies have demonstrated that the regulation of GBM CSCs is approached from inhibition of the DNA-damage checkpoint kinases CHK1 and CHK2 using small molecules^1^. Using this strategy indicated an improved radiotherapy for GBM CSCs. Another alternative strategy of GBM treatment is the induction of a differentiated state of CSCs. A reliable report has demonstrated that GBM CSCs could be differentiated into endothelial cells, which could suppress the vasculature development^44^. However, the tumor-derived endothelial cells exist at a small portion in GBM tumor mass and its clinical relevance is questionable^45^. Zheng and colleagues demonstrated that the p53 together with PTEN could regulate the Myc expression in human primary GBM^46^. These GBM-derived cells after Myc suppression induced the down-regulation of nestin expression while it differentiate into Tuj1- and GFAP-expressing neural cell types. In this study, we assessed the GBM cell population that remained in the neural progenitor state, in which the nestin-expressing GBM CSCs reside. Interestingly, this cell population was diminished by CG500354 treatment but was not altered by treatment with mimetic substances (Fig. 3B and Fig. 5B). These results show that CG500354 plays a better role in human primary GBM and functions even better than Forskolin and Rolipram with respect to its potential anti-cancer effects.

From our findings on the mechanism of action of CG500354, we argue that p53 is implicated in the phenotype changes of the GBM-derived cells. Several studies have commonly identified mutations of tumor suppressor genes, such as p53, PTEN and RB, in brain tumor^47^,^48^. As shown in Figures 2F and 3B, we determined that the level of p53 expression was significantly increased after CG500354 treatment and was followed by differentiation into neural subtypes. We used QIAGEN's Ingenuity Pathway Analysis (IPA, QIAGEN Redwood City, www.qiagen.com/ingenuity) software to analyze molecular interactions between PDE4D and p53 (Suppl. Fig. S4). Based on IPA, representative regulatory networks were constructed that PDE4D directly activates PKA and cAMP and indirectly activates p21 and p27 as well as p53.Another reason for the dual effects of CG500354 is that the PDE4D inhibitor CG500354 could affect the cAMP/CREB signaling pathway, which is widely known as a signal-dependent gene regulatory pathway that is involved in many biological functions, including the cell cycle, cell survival and cell differentiation^24^,^25^. In the cAMP-dependent signal transduction pathway, PDE4D plays a suppressive role, as it causes a decrease in the level of cAMP. In a recent report on the role of cAMP, PDE4 was indicated as the most important target for the induction of apoptosis and differentiation in leukemia cells^49^,^50^. In another study, PDE4D was demonstrated to be abundantly expressed in glioblastoma cells and its inhibitor Rolipram was found to induce tumor regression^21^. Likewise, our data in Figure 4 reveals that CG500354, Forskolin and Rolipram could regulate the level of cAMP and its downstream target CREB. Together with data described above, our results indicate that CG500354 up-regulates the cAMP/CREB signaling pathway activity and induces the differentiation of human primary GBM.

In conclusion, we have shown that the protein level of p53 and the extracellular level of cAMP are significantly increased upon CG500354 treatment. We further indicated that CG500354 can alter the phenotypes of human primary GBM cells from an oncogenic state into a less oncogenic and more differentiated state. During these tumor-suppressive events, p21- and p27-mediated growth arrest and CREB-mediated neural differentiation also occurred. Moreover, the effects of CG500354 on the neural differentiation of the cells were more significant with respect to its reduction of the nestin-expressing population than the mimetic substances Forskolin and Rolipram. Therefore, the novel small molecule CG500354, which targets PDE4D, might be important in the development of new drugs for human GBM.

## Methods

### Isolation of primary GBM cells

Following informed consent and in accordance with the appropriate institutional review boards, GBM was obtained from a patient undergoing surgery at the Samsung Medical Center (Seoul, Republic of Korea). Tumors were classified as GBM based on WHO criteria by examination of pathologists^51^. Within one hour of surgical resection, the tumor mass was mechanically and enzymatically dissociated into single cells. GBM was briefly maintained in NBE neurosphere culture medium: Neurobasal-A medium (Invitrogen, Carlsbad, CA, USA) supplemented with recombinant human basic fibroblast and epidermal growth factors (50 ng/ml each; Invitrogen), N2 and B27 supplements (Invitrogen), 2 mM glutamine (Invitrogen), 100 units/ml penicillin and 100 mg/ml streptomycin (Invitrogen). We used human primary cells from three different GBM origins: GBM559, GBM592 and GBM626.

### Chemicals

CG500354 (Patent No. 61/470884) was provided by Crystal Genomics, Inc. (Seongnam, Republic of Korea). Rolipram (C_16_H_21_NO_3_; 4-[3-(cyclopentyloxy)-4-methoxyphenyl]-2-pyrroli-dinone) and Forskolin (C_22_H_34_O_7_; 7β-acetoxy-8,13-epoxy-1α,6β,9α-trihydroxylabd-14-en-11-one) were purchased from Sigma Aldrich (Saint Louis, MO, USA). For the cell treatments, Rolipram and Forskolin were used at a concentration of 10 μM.

### Clonogenic assays

Dissociated sphere cells (5 × 10^5^) were cultured in ultra-low attachment dishes. The cells were treated with DMSO or CG500354 for 72 hours. The number of spheres with a diameter of >100 μm were counted randomly in triplicate cultures.

### Magnetic-activated cell sorting

To isolate the CD133-positive GBM-derived cells from the DMSO- or CG500354-treated cells, we resuspended the treated cells with 300 μl/10^8^ buffer (PBS with 0.5% FBS) and added 50 μl/10^8^ of FcR Blocking Reagent and 50 μl/10^8^ CD133 MicroBeads (#130-050-801, Miltenyi Biotec) for 30 minutes at 6 °C to 12 °C. Then, we isolated CD133-positive cells using through Column and stained with anti-CD133/2-PE antibody (Miltenyi Biotec) to further analyze.

### Flow cytometry analysis

GBM-derived cells were treated with DMSO or CG500354 for 72 hours and stained with an anti-CD133/2-PE antibody (#130-090-853, Miltenyi Biotec, Bergisch Gladbach, Germany) for 30 minutes. The CD133-stained GBM-derived cells were analyzed with a FACSCalibur flow cytometer (Becton Dickinson).

### cAMP measurement

GBM cells were treated with DMSO or CG500354 (3 μM/ml) for 72 hours. The cAMP level was measured from the collected supernatants using a cyclic AMP EIA Kit (#581001, Cayman Chemical Company, Ann Arbor, MI, USA) according to the manufacturer's instructions. The cAMP values were calculated using a multifunction microplate reader (Tecan, San Jose, CA, USA).

### siRNA inhibition study

To specifically inhibit PDE4D expression, siRNA-mediated gene knockdown studies were performed using commercial siRNAs targeting the PDE4D transcript (ON Target plus SMART pool, Dharmacon, Lafayette, CO, USA) along with a non-targeting siRNA (#D-001810-01, ON Target plus SMART pool, Dharmacon). The siRNA transfections were performed according to the manufacturer's instructions. Briefly, the cells were seeded at a concentration of 5 × 10^5^ per well in 6-well plates. When the cells reached 60–70% confluence, a mixture of 50 nM siRNA and transfection reagent was added to the cell culture medium without antibiotics. The cells were incubated for 24 hours before evaluating mRNA expression by RT-PCR analysis or for 48 hours before evaluating protein expression by western blot analysis.

### Quantitative real-time PCR (qRT-PCR)

The relative gene expression levels were calculated using the 2^−ΔΔCt^ method after normalization to the endogenous expression level of GAPDH. All the primers used in this study are listed in Supplemental Table S1.

### Western blot analysis

For western blot analysis, the following primary antibodies were used: mouse monoclonal anti-GFAP (ab4648; Abcam, Cambridge, UK), mouse monoclonal anti-Tuj1 (MMS435; Covance, Princeton, NJ, USA), mouse monoclonal anti-P53 (2524; Cell Signaling Technology Inc., Boston, MA, USA), rabbit monoclonal anti-p-CREB (9198; Cell Signaling Technology Inc.), rabbit monoclonal anti-p-PKA (5661; Cell Signaling Technology Inc.) and mouse monoclonal anti-GAPDH (MAB374; Millipore, Billerica, MA, USA).

### Immunocytochemistry

The primary antibodies used for immunocytochemistry were: mouse monoclonal anti-nestin (MAB5326; Millipore), mouse monoclonal anti-GFAP (ab4648; Abcam), mouse monoclonal anti-Tuj1 (MAB1195; R&D Systems, Minneapolis, MN, USA) and mouse monoclonal anti-P53 (2524; Cell Signaling Technology Inc.). Both fluorescent secondary antibody conjugates, goat anti-mouse Alexa Fluor 488 (Invitrogen) and goat anti-rabbit Alexa Fluor 488 (Invitrogen), were used at a 1:1000 dilution. DAPI was used for counterstaining. The coverslips were mounted with fluorescent mounting solution (DAKO, Carpinteria, CA, USA) and observed by confocal microscopy (Eclipse TE200, Nikon, Japan).

### Animal experiments

NOD-SCID mice were purchased from Jackson Laboratories (Bar Harbor, ME, USA). All experiments were performed in accordance with the guidelines and regulation, which were approved by the Institute of Laboratory Animals Resources (SNU-101013-5, Seoul National University, Republic of Korea). Four-week-old female mice were subcutaneously injected with 1 × 10^7^ GBM-derived cells, which were suspended in 0.1 ml PBS. When tumors appeared, the mice were randomly divided into two groups and treated daily with intraperitoneal injections of 3 mg/kg CG500354 (n = 4) or DMSO as the vehicle control (n = 3) for 10 days. The mice were sacrificed, and the tumors were separated from the host for histological evaluation.

### Statistical analysis

All experiments were conducted at least in triplicate, and the results are expressed as the mean ± SD. The statistical analyses were conducted using analysis of variance (ANOVA) followed by a Duncan's multiple range test or Student's t-test. The following *P* values were considered significant: *, *P* < 0.05; **, *P* < 0.01; ***, *P* < 0.001.

## Author Contributions

T.K. and S.C. wrote the main manuscript text and prepared all figures. S.Y. made substantial contributions to conception and design. T.S. and H.K. made substantial contributions to acquisition of data and analysis and interpretation of data. K.Y., I.H., S.R., J.C. and K.K. participated in revising the article critically for important intellectual content. K.K. gave final approval of the version to be submitted. All authors reviewed the manuscript.

## Supplementary Material

Supplementary Information Supplementary Information

## References

[b1] Bao S. *et al.* Glioma stem cells promote radioresistance by preferential activation of the DNA damage response. Nature 444, 756–760 (2006). 17051156 10.1038/nature05236

[b2] Liu G. *et al.* Analysis of gene expression and chemoresistance of CD133+ cancer stem cells in glioblastoma. Mol Cancer 5, 67 (2006). 17140455 10.1186/1476-4598-5-67 PMC1697823

[b3] Stupp R. *et al.* Effects of radiotherapy with concomitant and adjuvant temozolomide versus radiotherapy alone on survival in glioblastoma in a randomised phase III study: 5-year analysis of the EORTC-NCIC trial. Lancet Oncol 10, 459–466 (2009). 19269895 10.1016/S1470-2045(09)70025-7

[b4] Beck B. & Blanpain C. Unravelling cancer stem cell potential. Nat Rev Cancer 13, 727–738 (2013). 24060864 10.1038/nrc3597

[b5] Peh G. S., Lang R. J., Pera M. F. & Hawes S. M. CD133 expression by neural progenitors derived from human embryonic stem cells and its use for their prospective isolation. Stem Cells Dev 18, 269–282 (2009). 18651819 10.1089/scd.2008.0124

[b6] Carter D. A., Dick A. D. & Mayer E. J. CD133+ adult human retinal cells remain undifferentiated in Leukaemia Inhibitory Factor (LIF). BMC Ophthalmol 9, 1 (2009). 19236693 10.1186/1471-2415-9-1 PMC2649894

[b7] Uchida N. *et al.* Direct isolation of human central nervous system stem cells. Proc Natl Acad Sci U S A 97, 14720–14725 (2000). 11121071 10.1073/pnas.97.26.14720 PMC18985

[b8] Singh S. K. *et al.* Identification of human brain tumour initiating cells. Nature 432, 396–401 (2004). 15549107 10.1038/nature03128

[b9] Singh S. & Dirks P. B. Brain tumor stem cells: identification and concepts. Neurosurg Clin N Am 18, 31–38, viii (2007). 17244552 10.1016/j.nec.2006.10.014

[b10] Eramo A. *et al.* Identification and expansion of the tumorigenic lung cancer stem cell population. Cell Death Differ 15, 504–514 (2008). 18049477 10.1038/sj.cdd.4402283

[b11] Collins A. T., Berry P. A., Hyde C., Stower M. J. & Maitland N. J. Prospective identification of tumorigenic prostate cancer stem cells. Cancer Res 65, 10946–10951 (2005). 16322242 10.1158/0008-5472.CAN-05-2018

[b12] Reya T., Morrison S. J., Clarke M. F. & Weissman I. L. Stem cells, cancer, and cancer stem cells. Nature 414, 105–111 (2001). 11689955 10.1038/35102167

[b13] Galli R. *et al.* Isolation and characterization of tumorigenic, stem-like neural precursors from human glioblastoma. Cancer Res 64, 7011–7021 (2004). 15466194 10.1158/0008-5472.CAN-04-1364

[b14] Yuan X. *et al.* Isolation of cancer stem cells from adult glioblastoma multiforme. Oncogene 23, 9392–9400 (2004). 15558011 10.1038/sj.onc.1208311

[b15] Beier D. *et al.* CD133(+) and CD133(-) glioblastoma-derived cancer stem cells show differential growth characteristics and molecular profiles. Cancer Res 67, 4010–4015 (2007). 17483311 10.1158/0008-5472.CAN-06-4180

[b16] Lathia J. D. *et al.* Integrin alpha 6 regulates glioblastoma stem cells. Cell Stem Cell 6, 421–432 (2010). 20452317 10.1016/j.stem.2010.02.018 PMC2884275

[b17] Piccirillo S. G. *et al.* Bone morphogenetic proteins inhibit the tumorigenic potential of human brain tumour-initiating cells. Nature 444, 761–765 (2006). 17151667 10.1038/nature05349

[b18] de The H. & Chen Z. Acute promyelocytic leukaemia: novel insights into the mechanisms of cure. Nat Rev Cancer 10, 775–783 (2010). 20966922 10.1038/nrc2943

[b19] Nasr R. *et al.* Eradication of acute promyelocytic leukemia-initiating cells through PML-RARA degradation. Nat Med 14, 1333–1342 (2008). 19029980 10.1038/nm.1891

[b20] Guillemin M. C. *et al.* In vivo activation of cAMP signaling induces growth arrest and differentiation in acute promyelocytic leukemia. J Exp Med 196, 1373–1380 (2002). 12438428 10.1084/jem.20021129 PMC2193985

[b21] Goldhoff P. *et al.* Targeted inhibition of cyclic AMP phosphodiesterase-4 promotes brain tumor regression. Clin Cancer Res 14, 7717–7725 (2008). 19047098 10.1158/1078-0432.CCR-08-0827 PMC2615415

[b22] Yang L., Jackson E., Woerner B. M., Perry A., Piwnica-Worms D. & Rubin J. B. Blocking CXCR4-mediated cyclic AMP suppression inhibits brain tumor growth in vivo. Cancer Res 67, 651–658 (2007). 17234775 10.1158/0008-5472.CAN-06-2762

[b23] Moreno M. J., Ball M., Andrade M. F., McDermid A. & Stanimirovic D. B. Insulin-like growth factor binding protein-4 (IGFBP-4) is a novel anti-angiogenic and anti-tumorigenic mediator secreted by dibutyryl cyclic AMP (dB-cAMP)-differentiated glioblastoma cells. Glia 53, 845–857 (2006). 16586492 10.1002/glia.20345

[b24] Mayr B. M., Canettieri G. & Montminy M. R. Distinct effects of cAMP and mitogenic signals on CREB-binding protein recruitment impart specificity to target gene activation via CREB. Proc Natl Acad Sci U S A 98, 10936–10941 (2001). 11535812 10.1073/pnas.191152098 PMC58577

[b25] Mayr B. & Montminy M. Transcriptional regulation by the phosphorylation-dependent factor CREB. Nat Rev Mol Cell Biol 2, 599–609 (2001). 11483993 10.1038/35085068

[b26] Siddappa R. *et al.* cAMP/PKA pathway activation in human mesenchymal stem cells in vitro results in robust bone formation in vivo. Proc Natl Acad Sci U S A 105, 7281–7286 (2008). 18490653 10.1073/pnas.0711190105 PMC2387183

[b27] Sato K. *et al.* Regulation of osteoclast differentiation and function by the CaMK-CREB pathway. Nat Med 12, 1410–1416 (2006). 17128269 10.1038/nm1515

[b28] Zhao Q. *et al.* Rapid induction of cAMP/PKA pathway during retinoic acid-induced acute promyelocytic leukemia cell differentiation. Leukemia 18, 285–292 (2004). 14628075 10.1038/sj.leu.2403226

[b29] Lucchi S., Calebiro D., de Filippis T., Grassi E. S., Borghi M. O. & Persani L. 8-Chloro-cyclic AMP and protein kinase A I-selective cyclic AMP analogs inhibit cancer cell growth through different mechanisms. PLoS One 6, e20785 (2011). 21695205 10.1371/journal.pone.0020785 PMC3112188

[b30] Landis C. A., Masters S. B., Spada A., Pace A. M., Bourne H. R. & Vallar L. GTPase inhibiting mutations activate the alpha chain of Gs and stimulate adenylyl cyclase in human pituitary tumours. Nature 340, 692–696 (1989). 2549426 10.1038/340692a0

[b31] Kirschner L. S. *et al.* Mutations of the gene encoding the protein kinase A type I-alpha regulatory subunit in patients with the Carney complex. Nat Genet 26, 89–92 (2000). 10973256 10.1038/79238

[b32] Lania A., Mantovani G. & Spada A. G protein mutations in endocrine diseases. Eur J Endocrinol 145, 543–559 (2001). 11720871 10.1530/eje.0.1450543

[b33] Persani L. *et al.* Induction of specific phosphodiesterase isoforms by constitutive activation of the cAMP pathway in autonomous thyroid adenomas. J Clin Endocrinol Metab 85, 2872–2878 (2000). 10946896 10.1210/jcem.85.8.6712

[b34] Otmakhov N. *et al.* Forskolin-induced LTP in the CA1 hippocampal region is NMDA receptor dependent. J Neurophysiol 91, 1955–1962 (2004). 14702333 10.1152/jn.00941.2003

[b35] Romano A., Delorenzi A., Pedreira M. E., Tomsic D. & Maldonado H. Acute administration of a permeant analog of cAMP and a phosphodiesterase inhibitor improve long-term habituation in the crab Chasmagnathus. Behav Brain Res 75, 119–125 (1996). 8800648 10.1016/0166-4328(96)00179-9

[b36] Schneider H. H. Brain cAMP response to phosphodiesterase inhibitors in rats killed by microwave irradiation or decapitation. Biochem Pharmacol 33, 1690–1693 (1984). 6203537 10.1016/0006-2952(84)90295-8

[b37] Park S. J. *et al.* Resveratrol ameliorates aging-related metabolic phenotypes by inhibiting cAMP phosphodiesterases. Cell 148, 421–433 (2012). 22304913 10.1016/j.cell.2012.01.017 PMC3431801

[b38] Li Y. F. *et al.* Phosphodiesterase-4D knock-out and RNA interference-mediated knock-down enhance memory and increase hippocampal neurogenesis via increased cAMP signaling. J Neurosci 31, 172–183 (2011). 21209202 10.1523/JNEUROSCI.5236-10.2011 PMC3079568

[b39] Dowling-Warriner C. V. & Trosko J. E. Induction of gap junctional intercellular communication, connexin43 expression, and subsequent differentiation in human fetal neuronal cells by stimulation of the cyclic AMP pathway. Neuroscience 95, 859–868 (2000). 10670453 10.1016/s0306-4522(99)00411-x

[b40] Houslay M. D. & Adams D. R. PDE4 cAMP phosphodiesterases: modular enzymes that orchestrate signalling cross-talk, desensitization and compartmentalization. Biochem J 370, 1–18 (2003). 12444918 10.1042/BJ20021698 PMC1223165

[b41] Marko D., Pahlke G., Merz K. H. & Eisenbrand G. Cyclic 3′,5′-nucleotide phosphodiesterases: potential targets for anticancer therapy. Chem Res Toxicol 13, 944–948 (2000). 11080038 10.1021/tx000090l

[b42] Brown C. J., Lain S., Verma C. S., Fersht A. R. & Lane D. P. Awakening guardian angels: drugging the p53 pathway. Nat Rev Cancer 9, 862–873 (2009). 19935675 10.1038/nrc2763

[b43] Riley T., Sontag E., Chen P. & Levine A. Transcriptional control of human p53-regulated genes. Nat Rev Mol Cell Biol 9, 402–412 (2008). 18431400 10.1038/nrm2395

[b44] Ricci-Vitiani L. *et al.* Tumour vascularization via endothelial differentiation of glioblastoma stem-like cells. Nature 468, 824–828 (2010). 21102434 10.1038/nature09557

[b45] Rodriguez F. J., Orr B. A., Ligon K. L. & Eberhart C. G. Neoplastic cells are a rare component in human glioblastoma microvasculature. Oncotarget 3, 98–106 (2012). 22298889 10.18632/oncotarget.427 PMC3292896

[b46] Zheng H. *et al.* p53 and Pten control neural and glioma stem/progenitor cell renewal and differentiation. Nature 455, 1129–1133 (2008). 18948956 10.1038/nature07443 PMC4051433

[b47] Chow L. M. *et al.* Cooperativity within and among Pten, p53, and Rb pathways induces high-grade astrocytoma in adult brain. Cancer Cell 19, 305–316 (2011). 21397855 10.1016/j.ccr.2011.01.039 PMC3060664

[b48] Verhaak R. G. *et al.* Integrated genomic analysis identifies clinically relevant subtypes of glioblastoma characterized by abnormalities in PDGFRA, IDH1, EGFR, and NF1. Cancer Cell 17, 98–110 (2010). 20129251 10.1016/j.ccr.2009.12.020 PMC2818769

[b49] Karin M. Signal transduction from the cell surface to the nucleus through the phosphorylation of transcription factors. Curr Opin Cell Biol 6, 415–424 (1994). 7917334 10.1016/0955-0674(94)90035-3

[b50] Copsel S. *et al.* Multidrug resistance protein 4 (MRP4/ABCC4) regulates cAMP cellular levels and controls human leukemia cell proliferation and differentiation. J Biol Chem 286, 6979–6988 (2011). 21205825 10.1074/jbc.M110.166868 PMC3044954

[b51] Louis D. N. *et al.* The 2007 WHO classification of tumours of the central nervous system. Acta Neuropathol 114, 97–109 (2007). 17618441 10.1007/s00401-007-0243-4 PMC1929165

