# Supplementary material for: Growth arrest and forced differentiation of human primary glioblastoma multiforme by a novel small molecule
Source: Sci Rep. 2014 Jul 3;4:5546. doi: 10.1038/srep05546 (PMC4080225; doi:10.1038/srep05546)
Supplement: Supplementary Information [file srep05546-s1.doc]

**Growth arrest and forced differentiation of human primary glioblastoma multiforme by a novel small molecule**

Tae-Wook Kang^1,2,†^, Soon Won Choi^1,2,†^, Se-Ran Yang^3,†^, Tae-Hoon Shin^1,2^, Hyung-Sik Kim^1^, Kyung-Rok Yu^1^, In-Sun Hong^4,5^, Seonggu Ro^6^, Joong Myung Cho^6^, Kyung-Sun Kang^1,2,*^

1 Adult Stem Cell Research Center, College of Veterinary Medicine, Seoul National University, Seoul, Republic of Korea

2 BK21 PLUS Program for Creative Veterinary Science Research, Research Institute for Veterinary Science and College of Veterinary Medicine, Seoul National University

3 Department of Thoracic and Cardiovascular Surgery, Kangwon National University Hospital, School of Medicine, Kangwon National University, Chuncheon, Republic of Korea

4 Department of Molecular Medicine, Gachon University, Incheon, Republic of Korea

5 Lee Gil Ya Cancer and Diabetes Institute, Gachon University, Incheon, Republic of Korea

6 CrystalGenomics, Inc., Korea Bio Park, 700 Daewangpangyo-ro, Bundang-gu, Seongnam, Republic of Korea

† These authors contributed equally to this work.

* Corresponding author


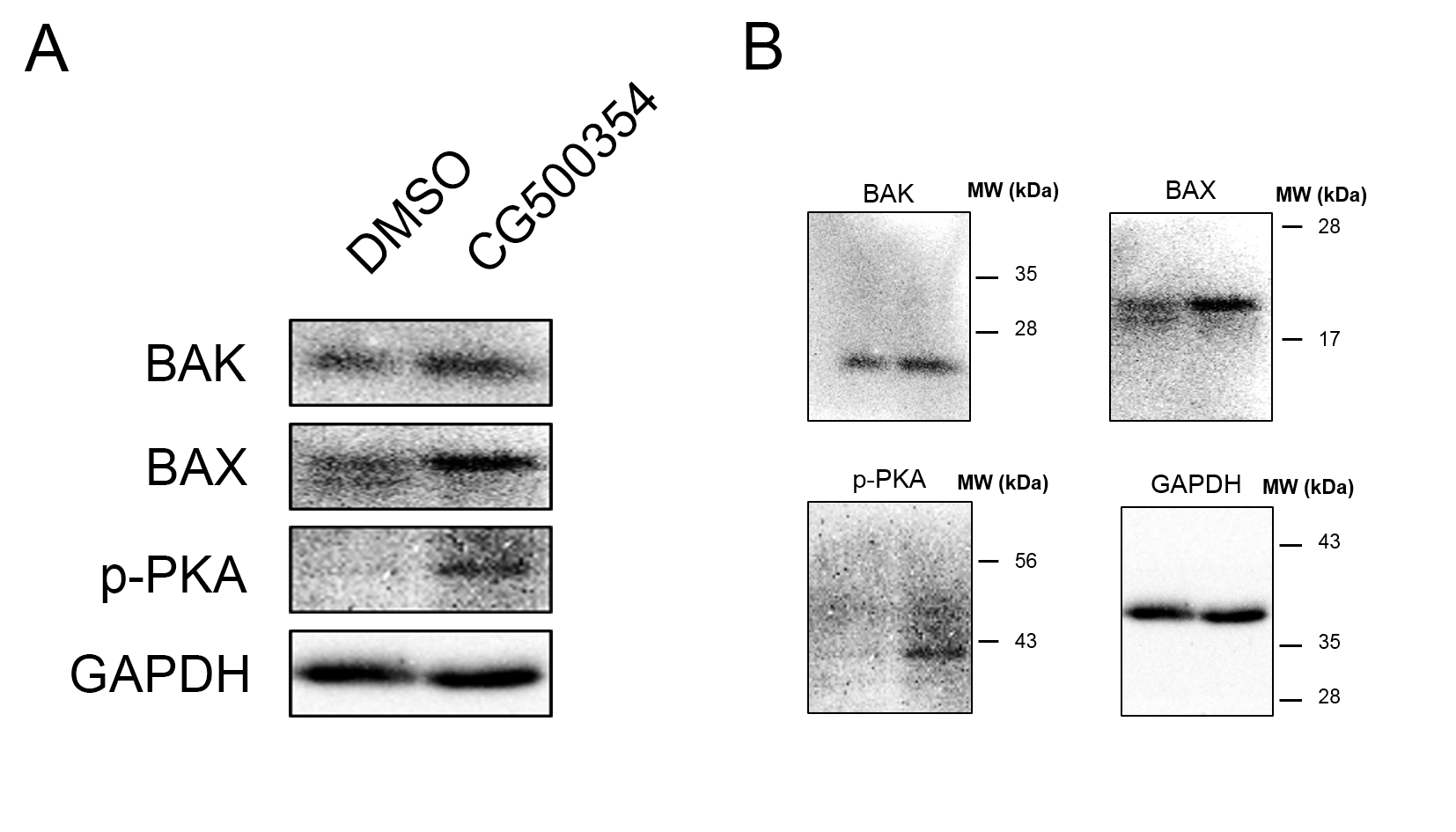


**Supplementary Figure S1**. (A) Protein expression of two apoptotic markers and phosphorylated PKA in GBM-derived cells after a CG500354 treatment. (B) The cropped blots are shown.


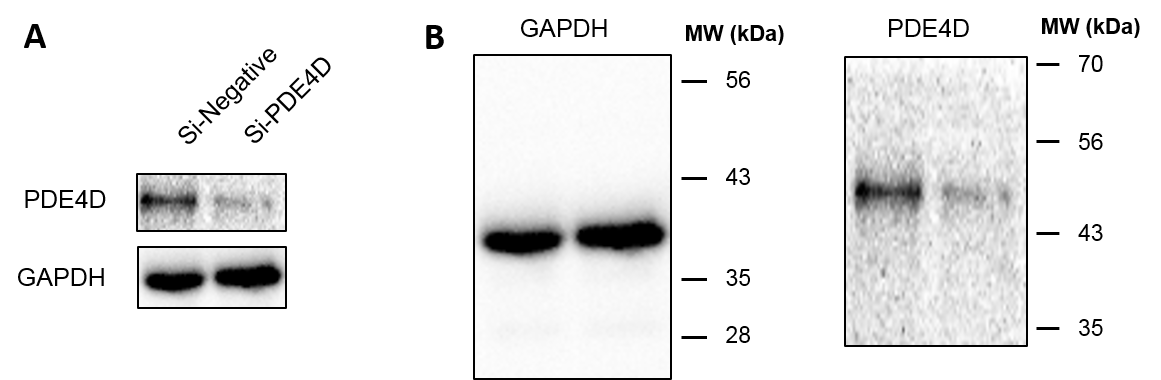


**Supplementary Figure S2**. (A) A representative western blot analysis shows knockdown of PDE4D protein expression in GBM-derived cells after a CG500354 treatment. (B) The cropped blots are shown.


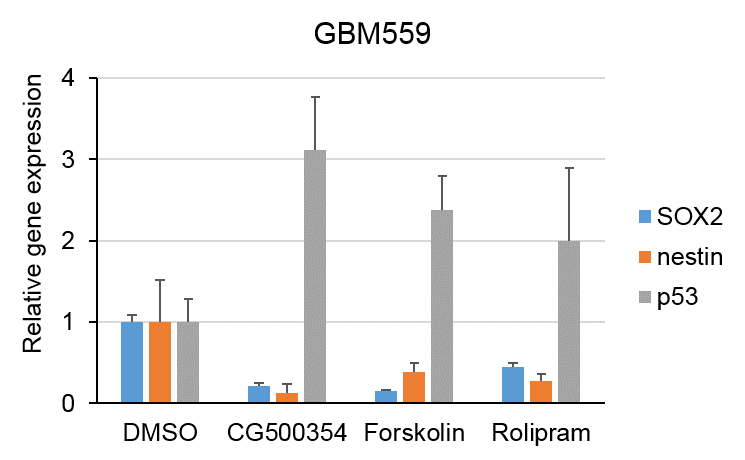

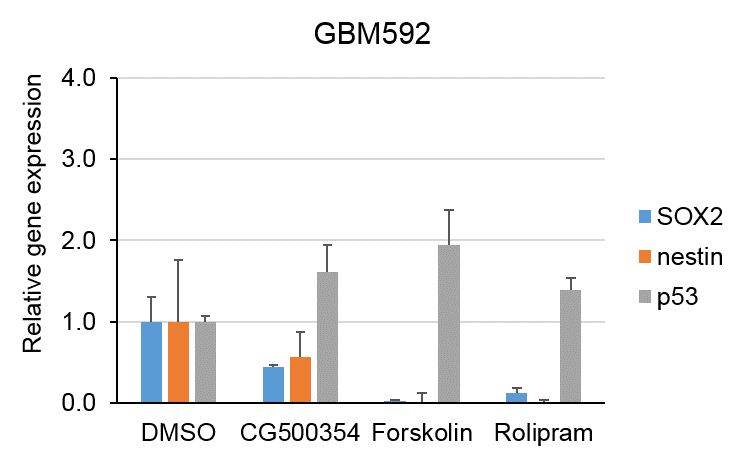

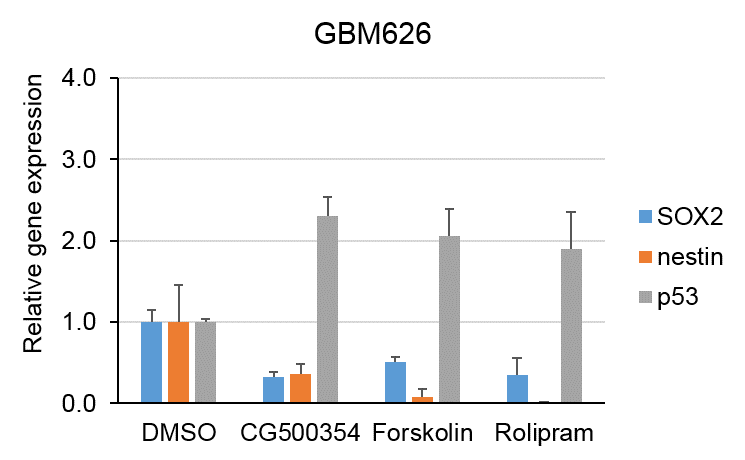


**Supplementary Figure S3**. Change of expression pattern of neural stem cell markers, SOX2 and nestin, as well as p53 in GBM cells of three different GBM origins: GBM559, GBM592 and GBM626. Quantitative RT-PCR analyses indicate relative gene expression levels in CG500354-, Forskolin- and Rolipram-treated GBM-derived cells.


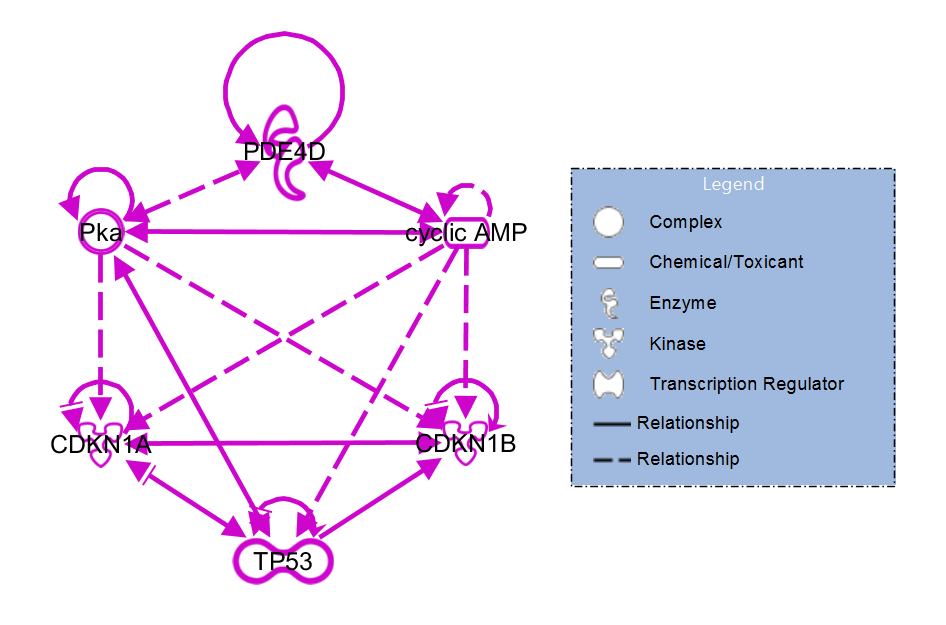


**Supplementary Figure S4**. (A) Ingenuity Pathway Analysis was performed on PDE4D, PKA (Pka), cAMP (cyclic AMP), p21 (CDKN1A), p27 (CDKN1B) and p53 (TP53). This panel shows the interactions between those six components.


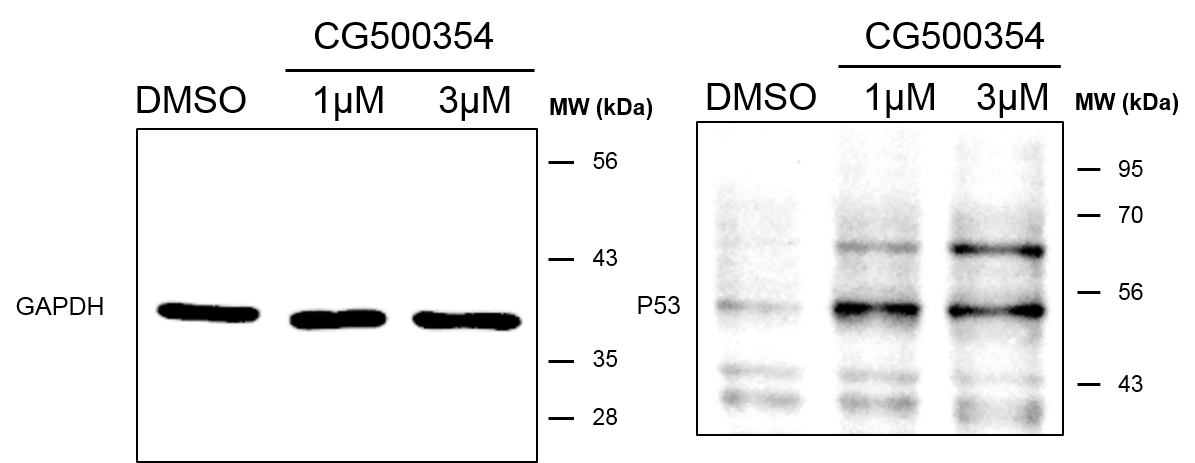


**Supplementary Figure S5**. Tumor Suppressive effect of CG500354 on human GBM. The cropped blots are used in the main figure (Figure 2D).


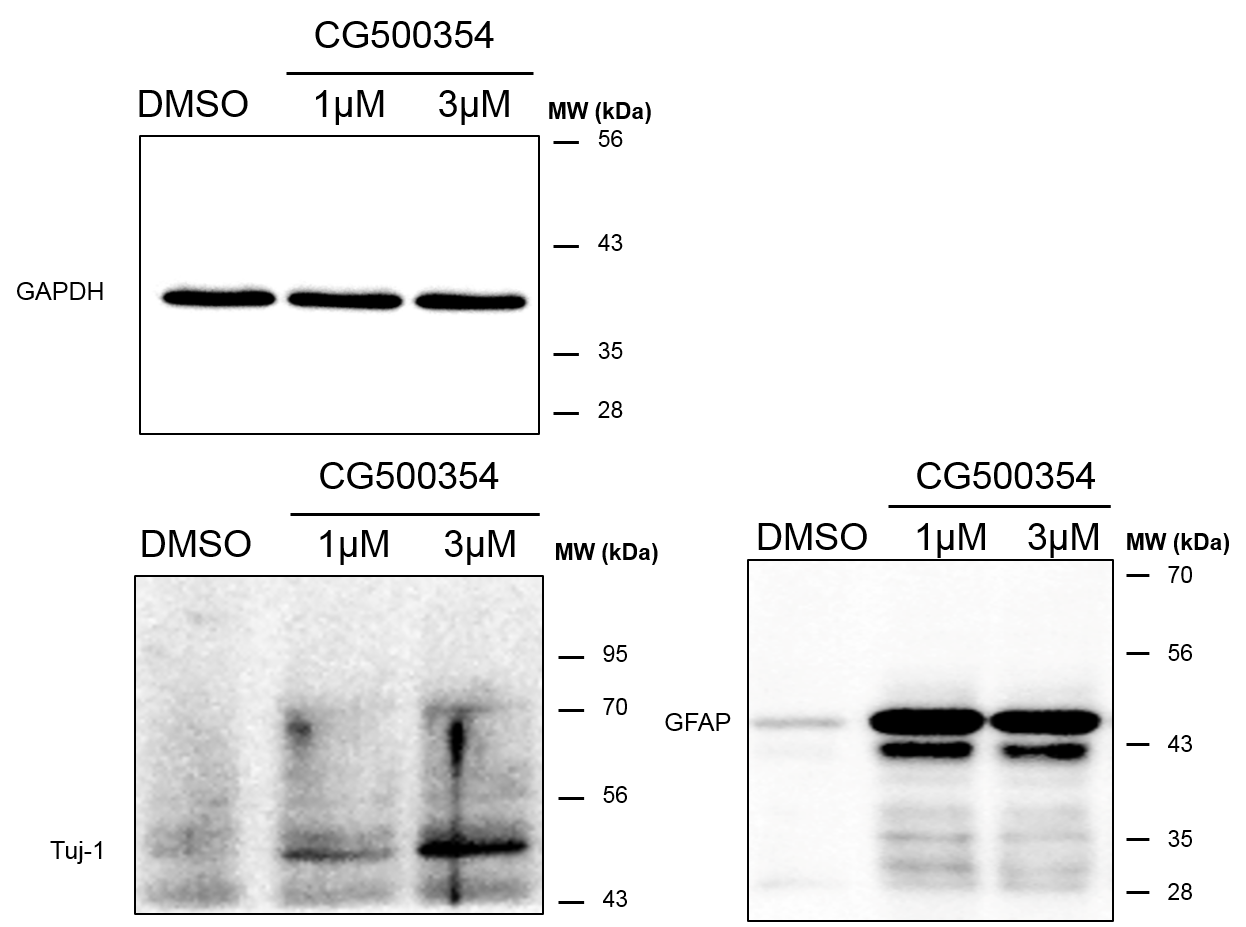


**Supplementary Figure S6**. Expression of neural differentiation markers after CG500354 treatment. The cropped blots are used in the main figure (Figure 3D).


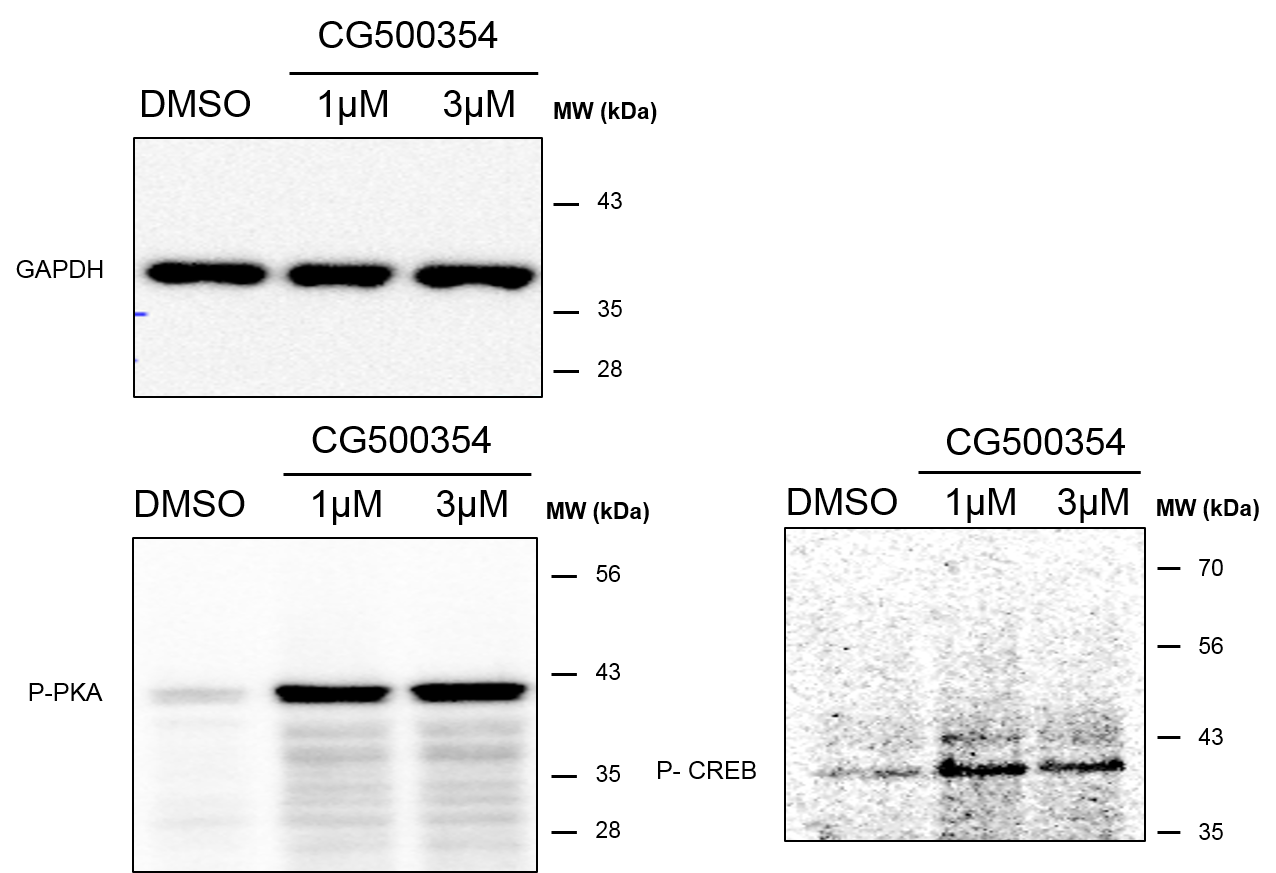


**Supplementary Figure S7**. Induction of gene expression of cAMP/CREB signaling pathway after CG500354 treatment. The cropped blots are used in the main figure (Figure 4B).


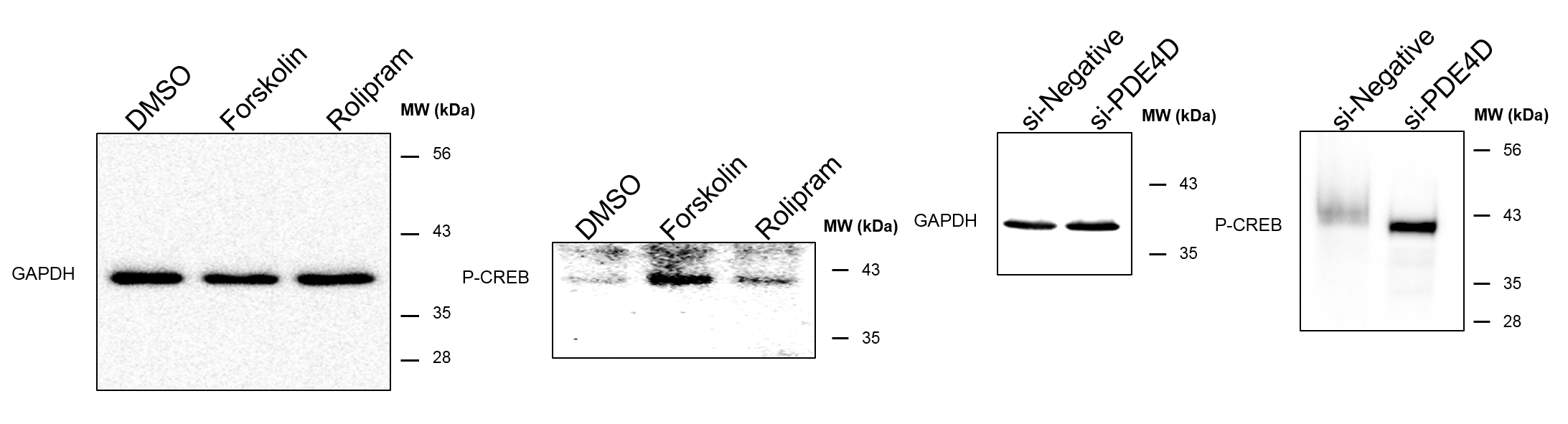


**Supplementary Figure S8**. Induction of gene expression of cAMP/CREB signaling pathway after both mimetic substances (Forskolin and Rolipram) and si-PDE4D treatment. The cropped blots are used in the main figure (Figure 4C).


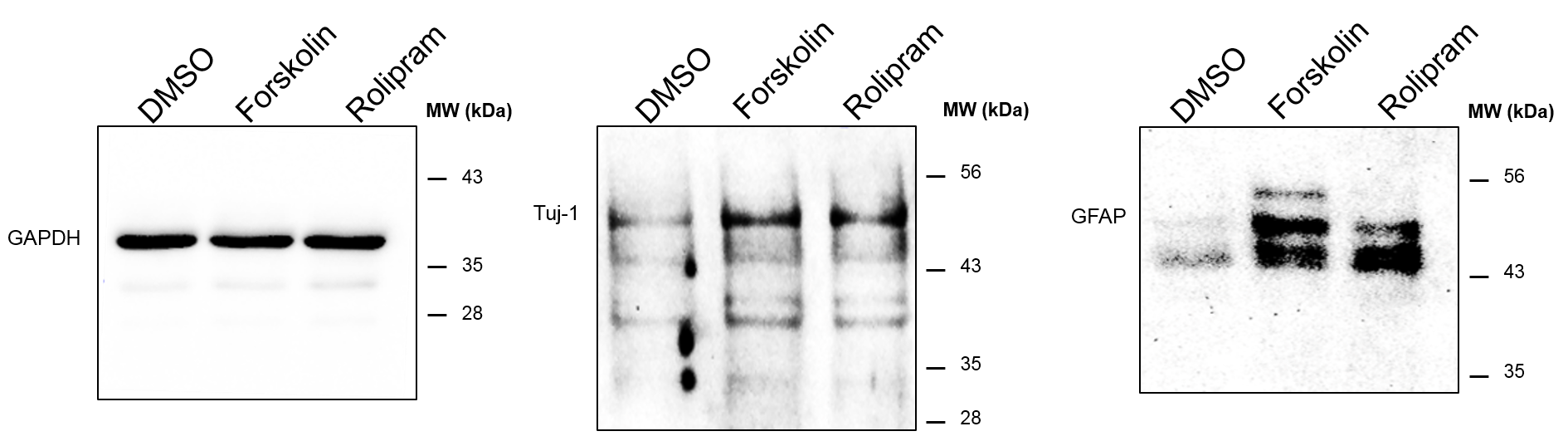


**Supplementary Figure S9**. Expression of neural differentiation markers after both mimetic substances (Forskolin and Rolipram) treatment. The cropped blots are used in the main figure (Figure 5A).


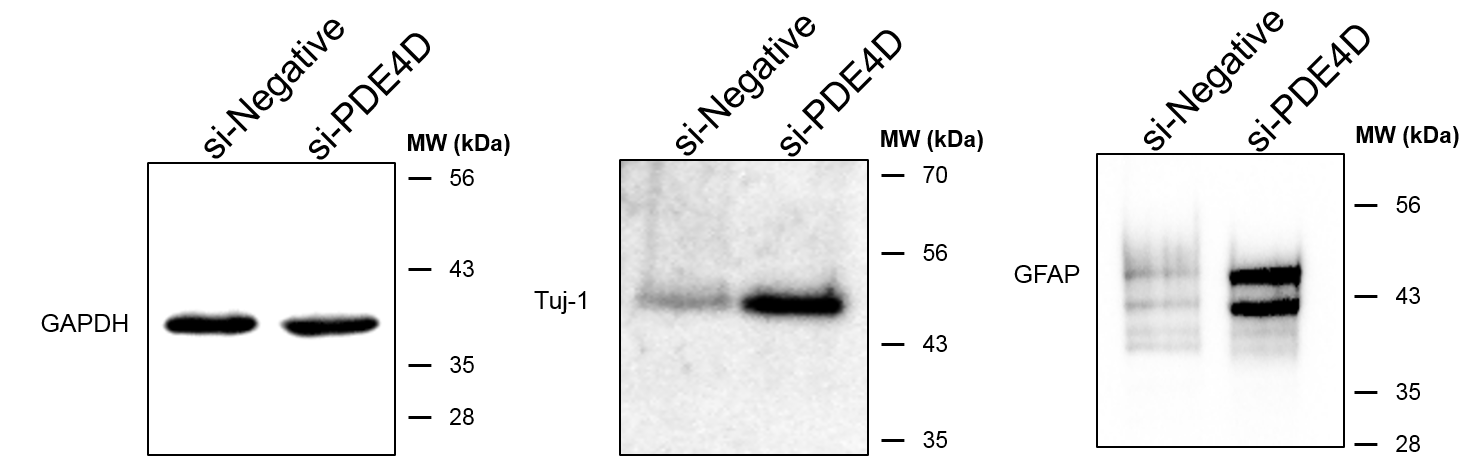


**Supplementary Figure S10**. Expression of neural differentiation markers after si-PDE4D treatment. The cropped blots are used in the main figure (Figure 5D).

| **Gene** | **Forward** | **Reverse** | **cDNA product(s) (bp)** | **gDNA product (bp)** |
| --- | --- | --- | --- | --- |
| Nestin | ACCTCAAGATGTCCCTCAGC | GAGCAAAGATCCAAGACGCC | 176 and 89 | 2112 |
| CCNA1 | GTCACTTGGGATGGAGACCG | GCATTGCTTCAGACTCCACG | 143 | 449bp |
| CCNB1 | GCCACGAACAGGCCAATAAG | CGCCTGCCATGTTGATCTTC | 250 | 874bp |
| CCND1 | CCCTCGGTGTCCTACTTCAAA | TGGAGGGCGGATTGGAAATG | 487 | X |
| CCND3 | AGACTGGCTCTGTTCGGATG | GCAAGACAGGTAGCGATCCA | 238 | X |
| p21 | AGTCAGTTCCTTGTGGAGCC | CATTAGCGCATCACAGTCGC | 184 | X |
| p27 | ACCTGCAACCGACGATTCTT | GTCCATTCCATGAAGTCAGCG | 252 | X |
| p53 | TGTGACTTGCACGTACTCCC | ACCATCGCTATCTGAGCAGC | 199 | X |
| GAPDH | CATGAGAAGTATGACAACAGCCT | AGTCCTTCCACGATACCAAAGT | 185 | X |

**Supplementary Table S1.** Primer list
